# Supplementary material for: Improving Patient Outcomes While Reducing Empirical Treatment with Multiplex-Polymerase-Chain-Reaction/Pooled-Antibiotic-Susceptibility-Testing Assay for Complicated and Recurrent Urinary Tract Infections
Source: Diagnostics (Basel). 2023 Sep 26;13(19):3060. doi: 10.3390/diagnostics13193060 (PMC10573050; doi:10.3390/diagnostics13193060)
Supplement: Supplementary file 1 [file diagnostics-13-03060-s001.zip › diagnostics-2589124-supplementary.pdf]

diagnostics

MDPI

Supplemental Materials

Study Item or Activity	Screening: Day 1	Index Visit: Day 1	Follow Up: Days 2-14	Follow Up: 30 Days	Comments	Completed By:
Inclusion / Exclusion Criteria Check<br>&<br>Informed Consent	X				Full inclusion and exclusion criteria are described in Supplemental Table S2.<br>The study obtained Western IRB approval (20214705) and was conducted in accordance with the Declaration of Helsinki. The IRB determined that the study protocol met the requirements for a partial waiver of authorization and only verbal patient informed consent was needed. All subjects provided verbal informed consent prior to enrollment.	Participants, Participating Investigator(s) & Research Coordinator
Urine Sample Collection		X			Urine collection methods included midstream voided clean-catch or catheterization using a sterile technique. There were no significant differences in rates of each collection method between test arms (supplemental table S3).	Participants
Arm Assignment		X			Each subject's assignment to one of the urine test arms was ordered at the discretion of the treating clinician.	Participating Investigator(s)
Urine Sample Delivery to Lab		X			Lab receives urine samples within 48 hours of collection time	Research Coordinator & Courier
Patient Baseline Survey		X			The baseline survey included questions evaluating baseline symptom severity and treatment information. The symptom portion of the survey used a validated American English Acute Cystitis Symptom Score (ACSS) Questionnaire, asking patients to evaluate the severity of typical UTI symptoms. The ACSS Questionnaire is validated using 5 different criteria to define the presence of UTI symptoms. Two of those criteria use subsets of symptoms defined by regulatory bodies: the European Medicines Agency (EMA) and the Food and Drug Administration (FDA). For this US-based study, the criterion based on the 4 UTI symptoms defined by the FDA Guidance for uncomplicated UTI (urinary frequency, urinary urgency, dysuria, and suprapubic pain) was chosen. The severity of each of the symptoms were scored from 0 to 3: no (0), mild (1), moderate (2), and severe (3). Only patients with at least one of the 4 FDA symptoms at baseline were included in the analyses.	Participants
Demographics and Medical History Form		X			See also the Treatment Decision Survey and the Patient Daily Survey sections below…	Participating Investigator(s) & Research Coordinator

Laboratory Processing and Testing of Urine Sample			X		Dipstick urinalysis may be ordered and performed separately by the healthcare provider.<br><br>Microbial identification and quantitation/antibiotic sensitivity results are delivered within 3-5 days for SUC or 24 hours for M-PCR/P-AST (after receipt by the lab).	Study Site Clinical Laboratories<br><br>and Pathnostics Laboratory
Patient Daily Survey			X		The Patient Daily Survey also included treatment information as reported by the participant.	Participants
Treatment Decision Survey		X	X		"Treated" was defined as treated with antimicrobial agents, including antibiotic and anti-fungal drugs, between day 1 and day 14. Study subjects who did not receive any medication, or who only received medication other than antimicrobial agents, were defined as "Untreated".<br><br>For the treated subjects, if the healthcare provider indicated the use of empirical treatment via the responses on the Demographics and Medical History Form and the Treatment Decision Survey, the subjects were categorized as "empirically treated". If the healthcare provider did not indicate the use of empirical treatment, the subjects receiving antimicrobial agents were categorized as "directly treated" based upon the test result reviewed by the clinician, which was determined by study arm.	Participating Investigator(s)
30 Day Survey				X	Clinical outcomes evaluated in this analysis were based on patient's responses to the clinical outcome questions on the day 30 follow-up survey. These questions included:<br>• "Have you been diagnosed with another UTI? Yes or No"<br>• "How many times have you gone back or seen another healthcare provider for UTI-related issues? 0, 1, 2, 3 or more"<br>• "How many times have you been hospitalized due to UTI-related issues? 0, 1, 2, 3 or more"<br>• "How many times have you visited the emergency department due to UTI-related issues? 0, 1, 2, 3 or more"<br>• "How many times have you visited urgent care due to UTI-related issues? 0, 1, 2, 3 or more".<br><br>Based on patients' responses to these questions, differences in the following three negative outcomes were measured:<br>1. a new UTI diagnosis since the index visit<br>2. a new visit to a medical provider for UTI symptoms<br>3. a UTI-related hospitalization or visit to UC or the ER<br><br>The composite negative outcome was defined as patients who had any one or more of these three negative outcomes. Each variable was analyzed separately, and the composite outcome was coded as a yes/no variable. The comparisons of each of the individual negative outcomes were also explored in the study.	Participants

Supplemental Table S1. Study Activities

Inclusion	Exclusion
Able to provide Informed Consent in English or Spanish	Failure to meet inclusion criteria (including those unable to provide Informed Consent)
Over 18 years of age	Inability to independently provide symptom data via an email-accessed online survey
Male or Female (no predetermined quotas or ratios for participation)	Special Populations: pregnant or incarcerated individuals
Presenting to a urologist or urogynecologist in an outpatient setting with a history of cUTI and clinically suspected active UTI (e.g., symptomatic with urinalysis positive for leukocyte esterase or nitrites)	Complicating conditions: currently receiving radiotherapy; bladder/urologic cancer; chronic pelvic pain; untreated overactive bladder; antibiotic use (other than for UTI); chronic (≥ 10 days) indwelling catheters; self-catheterization; or urinary diversion at the time of enrollment
Requires microbial testing according to clinician judgment	Clinician deems microbial testing unnecessary

*Definition of cUTI: UTI is considered complicated when the individual has one or more risk factors that predispose to higher treatment failure and poor outcomes. These poor outcomes include persistence of UTI, increasing severity, or occurrence of complications such as urosepsis, recurrence, and perinephric abscess.
Examples of cUTIs include:
UTIs in the elderly population due to increased chances of comorbidities and immune compromised state
Recurrent UTIs, which is defined as the occurrence of ≥2 symptomatic episodes within 6 months or ≥3 symptomatic episodes within 12 months
UTI in patients with anatomic or functional pathology affecting the urinary tract, such as an obstruction, hydronephrosis, renal tract calculi, or colovesical fistula
UTIs occurring due to an immune compromised state, such as steroid use, post chemotherapy, diabetes, and HIV
UTIs caused by atypical microorganisms or multi-drug resistant microorganisms. Typical UTI-causing microorganisms include *E. coli, P. aeruginosa*, several species within the Enterobacteriaceae family (Proteus and Klebsiella), and a few gram-positive bacteria, such as *Staphylococcus saprophyticus* and *Enterococcus faecalis*, as well as fungi, such as *Candida* sp75.
UTI in male: UTIs occurring despite the presence of anatomical protective measures as part of the male urinary tract anatomy are cUTI
UTI in patients with history of prior radiotherapy to the abdomen or pelvis
UTIs occurring after instrumentation, nephrostomy tubes, ureteric stents, suprapubic tubes or Foley catheters
UTI in patients with the history of recurrent UTI
UTIs in renal transplant patients
UTIs in patients with impaired renal function
UTIs following prostatectomies or radiotherapy

Supplemental Table S2. Study Inclusion and Exclusion Criteria

All unmatched (*N* = 577)

	M-PCR/P-AST (*n* = 429)	SUC (*n* = 148)	*p*-value
			0.21
Voided	425 (99.1%)	144 (97.3%)	
Catheter-Collected	4 (0.9%)	4 (2.7%)	

All matched

	M-PCR/P-AST (n = 252)	SUC (n = 146)	*p*-value
			0.47
Voided	248 (98.4%	142 (97.3%)	
Catheter-Collected	4 (1.6%)	4 (2.7%)	

≥60 unmatched

	M-PCR/P-AST (n = 325)	SUC (n = 91)	*p*-value
			1.00
Voided	322 (99.1%)	90 (98.9%)	
Catheter-Collected	3 (0.9%)	1 (1.1%)	

≥60 matched

	M-PCR/P-AST (n = 167)	SUC (n = 91)	*p*-value
			1.00
Voided	164 (98.2%)	90 (98.9%)	
Catheter-Collected	3 (1.8%)	1 (1.1%)	

Supplemental Table S3. Urine Specimen Collection Methods

Microorganism	Bacterial Cell Wall (gram-negative[a] or gram-positive[a])	Growth (fastidious[b] or non-fastidious[b])	Classification (classical[c] or emerging[c])
Acinetobacter	gram-negative	Non-fastidious	emerging
Actinotignum schaalii	gram-positive	fastidious	emerging
Aerococcus urinae	gram-positive	fastidious	emerging
Alloscardovia	gram-positive	fastidious	emerging
Candida albicans	NA - yeast	NA - yeast	classical
Candida auris	NA - yeast	NA - yeast	classical
Candida glabrata	NA - yeast	NA - yeast	classical
Candida parapsilosis	NA - yeast	NA - yeast	classical
*Citrobacter freundii**	gram-negative	Non-fastidious	classical
*Citrobacter koseri**	gram-negative	Non-fastidious	classical
Corynebacterium riegelii	gram-positive	fastidious	emerging
*Enterococcus faecalis**	gram-positive	Non-fastidious	classical
Enterococcus faecium	gram-positive	Non-fastidious**	classical
*Escherichia coli**	gram-negative	Non-fastidious	classical
Gardnerella vaginalis	gram-positive	fastidious	emerging
*Klebsiella oxytoca**	gram-negative	Non-fastidious	classical
*Klebsiella pneumoniae**	gram-negative	Non-fastidious	classical
*Morganella morganii**	gram-negative	Non-fastidious	classical
Mycoplasma hominis	NA - no cell wall	fastidious	emerging
*Pantoea agglomerans**	gram-negative	Non-fastidious**	emerging
*Proteus mirabilis**	gram-negative	Non-fastidious	classical
*Providencia stuartii**	gram-negative	Non-fastidious	classical
Pseudomonas	gram-negative	Non-fastidious	classical
*Serratia marcescens**	gram-negative	Non-fastidious	classical
*Staphylococcus aureus**	gram-positive	Non-fastidious	classical
Streptococcus	gram-positive	Non-fastidious	classical
Ureaplasma urealyticum	NA - No cell wall	fastidious	emerging
**Coagulase-negative *Staphylococci* (CoNS)* [*Staphylococcus epidermidis, Staphylococcus haemolyticus, Staphylococcus lugdunenesis, Staphylococcus saprophyticus*]**	gram-positive	Non-fastidious	emerging
**Viridans group *Streptococci* (VGS)**	gram-positive	Non-fastidious	emerging

Microorganism	Bacterial Cell Wall (gram-negative[a] or gram-positive[a])	Growth (fastidious[b] or non-fastidious[b])	Classification (classical[c] or emerging[c])
**[*Streptococcus anginosus, Streptococcus oralis, Streptococcus pasteuranus]***			
***Enterobacter* group* [*Klebsiella aerogenes* (formally known as *Enterobacter aerogenes*), *Enterobacter cloacae*]**	gram-negative	Non-fastidious	classical

*When detected, will be followed up with P-AST

**Not included in P-AST

[#]References for the role of each organisms as pathogens available upon request

[a]gram-positive vs gram-negative: gram-positive = bacteria that give a positive result in the Gram stain test, which uses crystal violet dye to categorize organisms based on the thickness of the peptidoglycan layer of the cell wall; gram-negative = bacteria that give a negative result in the Gram stain test

[b]fastidious vs non-fastidious: fastidious = microorganism that has complex or particular nutritional requirements; non-fastidious = microorganism with simple growth requirements met by standard urine culture conditions; non-fastidious = microorganism with simple growth requirements met by standard urine culture conditions

[c]classical vs emerging: classical = pathogens traditionally associated with UTI; emerging = microorganisms being newly recognized as potential or confirmed uropathogens

Supplemental Table S4. Microorganisms Targeted by Probes and Primers for the M-PCR Assay

Gene symbol	ThermoFisher (TF) Assay ID	Gene Name	CARD Link	Drug Class(es)	Resistance Mechanism
TEM	Pa04646128_s1	subclass B1 metallo-β-lactamase IMP (blaIMP)	https://card.mcmaster.ca/ontology/36023	Cephalosporin Penam Penem	Antibiotic Inactivation[a]
mecA	Ba04230908_s1	PBP2a family β-lactam-resistant peptidoglycan transpeptidase MecA (mecA)	https://card.mcmaster.ca/ontology/36911	Penicillin	Antibiotic Target Replacement[b]
A*mpC*	Ba04646117_s1	cephalosporin-hydrolyzing class C β-lactamase (blaACT)	https://card.mcmaster.ca/ontology/36215	β -lactam	Antibiotic Inactivation[a]
FOX	Ba04646126_s1	cephalosporin-hydrolyzing class C β-lactamase FOX (blaFOX)	https://card.mcmaster.ca/ontology/36206	Cephalosporin Cephamycin	Antibiotic Inactivation[a]
ACC	Ba04646144_s1	cephalosporin-hydrolyzing class C beta-lactamase ACC (blaACC)	https://card.mcmaster.ca/ontology/36212	Monobactam Cephalosporin Penam	Antibiotic Inactivation[a]
DHA	Ba04646120_s1	class C β -lactamase DHA (blaDHA)	https://card.mcmaster.ca/ontology/36207	Cephalosporin Cephamycin	Antibiotic Inactivation[a]
MOX/CMY	Ba04646156_s1	CMY-1/MOX family class C β -lactamase CMY(blaMOX)	https://card.mcmaster.ca/ontology/36226	β-lactam	Antibiotic Inactivation[a]

BIL/LAT/CMY	Ba04646135_s1	class C β -lactamase CMY (blaCMY)	https://card.mcmaster.ca/ontology/42866 https://card.mcmaster.ca/ontology/36208	Cephamycin_ Cephalosporin	Antibiotic Inactivation[a]
IMP-1 group	Ba04646131_s1	subclass B1 metallo-β-lactamase IMP (blaIMP)	https://card.mcmaster.ca/ontology/36029	Carbapenem Cephalosporin Cephamycin Penam Penem	Antibiotic Inactivation[a]
IMP-16	Ba04646116_s1	subclass B1 metallo-β-lactamase IMP (blaIMP)	https://card.mcmaster.ca/ontology/38607	Cephamycin Cephalosporin Penam Carbapenem Penem	Antibiotic Inactivation[a]
IMP-7	Ba04646158_s1	subclass B1 metallo-β-lactamase IMP(blaIMP)	https://card.mcmaster.ca/ontology/38598	Penem Penam Cephamycin Cephalosporin Carbapenem	Antibiotic Inactivation[a]
OXA-23	Ba04646139_s1	OXA-23 family carbapenem-hydrolyzing class D β-lactamase OXA (blaOXA)	https://card.mcmaster.ca/ontology/37818	Penem Penam Cephamycin Cephalosporin Carbapenem	Antibiotic Inactivation[a]
OXA-72	Ba04646118_s1	OXA-24 family carbapenem-hydrolyzing class D β-lactamase (blaOXA)	https://card.mcmaster.ca/ontology/38105	Cephalosporin Carbapenem Penam	Antibiotic Inactivation[a]
OXA-40	Ba04646143_s1	OXA-51 family carbapenem-	https://card.mcmaster.ca/ontology/37819	Cephalosporin Carbapenem Penam	Antibiotic Inactivation[a]

		hydrolyzing class D β-lactamase (blaOXA)		Monobactam	
blaOXA-48	Ba04930816_s1	OXA-48 family class D β-lactamase OXA (blaOXA)	https://card.mcmaster.ca/ontology/38182	Carbapenem Penam Cephalosporin	Antibiotic Inactivation[a]
VIM	Ba04646155_s1	subclass B1 metallo-β-lactamase VIM(blaVIM)	https://card.mcmaster.ca/ontology/36030	Penem Penam Cephamycin Cephalosporin Carbapenem	Antibiotic Inactivation
KPC	Ba04646152_s1	carbapenem-hydrolyzing class A β-lactamase KPC(blaKPC)	https://card.mcmaster.ca/ontology/36198	Cephalosporin Carbapenem Penam Monobactam	Antibiotic Inactivation[a]
CTX-M group 1	Ba04646149_s1	class A extended-spectrum β-lactamase CTX-M (blaCTX-M)	https://card.mcmaster.ca/ontology/36025	Cephalosporin	Antibiotic Inactivation[a]
CTX-M group 2	Ba04646142_s1	class A extended-spectrum β-lactamase CTX-M(blaCTX-M)	https://card.mcmaster.ca/ontology/36025	Cephalosporin	Antibiotic Inactivation[a]
CTX-M group 9	Ba04646127_s1	class A extended-spectrum β-lactamase CTX-M (blaCTX-M)	https://card.mcmaster.ca/ontology/36025	Cephalosporin	Antibiotic Inactivation[a]
CTX-M group 8/25	Ba04646154_s1	class A extended-spectrum β-lactamase CTX-M (blaCTX-M)	https://card.mcmaster.ca/ontology/36025	Cephalosporin	Antibiotic Inactivation[a]

OXA-1	Pa04646133_s1	OXA-1 family class D β-lactamase (blaOXA)	https://card.mcmaster.ca/ontology/37796	Carbapenem<br>Penam<br>Cephalosporin	Antibiotic Inactivation[a]
GES	Ba04646151_s1	class A β-lactamase GES(blaGES)	https://card.mcmaster.ca/ontology/36205	Carbapenem<br>Penam<br>Cephalosporin	Antibiotic Inactivation[a]
PER-1	Pa04646140_s1	class A extended-spectrum β-lactamase PER(blaPER)	https://card.mcmaster.ca/ontology/38763	Cephalosporin<br>Carbapenem<br>Penam<br>Monobactam<br>Penem	Antibiotic Inactivation[a]
PER-2	Pa04646157_s1	class A extended-spectrum β-lactamase PER (blaPER)	https://card.mcmaster.ca/ontology/44597	Cephalosporin<br>Carbapenem<br>Penam<br>Monobactam<br>Penem	Antibiotic Inactivation[a]
SHV	Ba04646134_s1	class A β-lactamase SHV (blaSHV)	https://card.mcmaster.ca/ontology/36024	Cephalosporin<br>Carbapenem<br>Penam	Antibiotic Inactivation[a]
VEB	Ba04646153_s1	class A extended-spectrum β-lactamase (blaVEB)	https://card.mcmaster.ca/ontology/36182	Cephalosporin<br>Monobactam	Antibiotic Inactivation[a]

QnrA	Ba04646160_s1	quinolone resistance pentapeptide repeat protein QnrA2 (qnrA)	https://card.mcmaster.ca/ontology/39142	Fluoroquinolone	Antibiotic Target Protection[c]
QnrS	Ba04646145_s1	quinolone resistance pentapeptide repeat protein QnrS9 (qnrS)	https://card.mcmaster.ca/ontology/39224	Fluoroquinolone	Antibiotic Target Protection[c]
VanA1	Ba04646159_s1	D-alanine--(R)-lactate ligase VanA (vanA)	https://card.mcmaster.ca/ontology/36019	Glycopeptide	Antibiotic Target Alteration[d]
VanA2	Ba04646147_s1	D-alanine--(R)-lactate ligase VanA (vanA)	https://card.mcmaster.ca/ontology/36019	Glycopeptide	Antibiotic Target Alteration[d]
VanB	Pa04646150_s1	D-alanine--(R)-lactate ligase VanB (vanB)	https://card.mcmaster.ca/ontology/36022	Glycopeptide	Antibiotic Target Alteration[d]

[#]References available upon request

[a]Antibiotic Inactivation - Chemically altering the antibiotic (Ex: via hydrolysis of the chemically reactive β-lactam ring)

[b]Antibiotic Target Replacement - Replacement or substitution of antibiotic action target

[c]Antibiotic Target Protection - Protection of antibiotic action target from antibiotic binding

[d]Antibiotic Target Alteration - Mutational alteration or enzymatic modification of antibiotic target

Supplemental Table S5. Antibiotic Resistance Genes Targeted by Probes and Primers for the M-PCR Assay

Antibiotic Name	Drug Class
Ampicillin	Aminopenicillin
Ampicillin/Sulbactam	Aminopenicillin - β-lactamase Inhibitor Combination
Amoxicillin/Clavulanate	Aminopenicillin - β-lactamase Inhibitor Combination
Cefazolin	Cephalosporin
Cefaclor	Cephalosporin
Cefepime	Cephalosporin
Cefoxitin	Cephalosporin
Ceftazidime	Cephalosporin
Ceftriaxone	Cephalosporin
Ciprofloxacin	Fluoroquinolone
Fosfomycin	A member of a novel class of Phosphonic antibiotics
Gentamicin	Aminoglycoside
Levofloxacin	Fluoroquinolone
Meropenem	β-lactam
Nitrofurantoin	Nitrofuran Derivative
Piperacillin/Tazobactam	β-lactam antibiotic of the ureidopenicillin class - β-lactamase Inhibitor Combination
Trimethoprim/Sulfamethoxazole	Antibacterial Folate Antagonist - Other Combination
Tetracycline	Tetracycline antibiotic family
Vancomycin	Glycopeptide

[#]References for the role of each organisms as pathogens available upon request

Supplemental Table S6. Antibiotics Tested in the P-AST Assay

	All (N = 577)			≥ 60 (n = 416)		
	SUC (n = 148)	M-PCR/ P-AST (n = 429)	p-value	SUC (n = 91)	M-PCR/ P-AST (n = 325)	p-value
Age			0.008			0.56
Mean (SD)	62.7 (14.9)	66.4 (14.4)		72.3 (7.6)	72.8 (7.4)	
Median (Min, Max)	64.8 (20.6, 96.0)	69.4 (18.9, 95.5)		72.2 (60.3, 96.0)	72.1 (60.2, 95.5)	
Sex			0.11			0.07
Male	44 (29.7%)	163 (38.0%)		29 (31.9%)	138 (42.5%)	
Female	104 (70.3%)	266 (62.0%)		62 (68.1%)	187 (57.5%)	
Baseline Symptom Score	5.0 (2.6)	4.7 (2.4)	0.13	5.4 (2.6)	4.6 (2.4)	0.006

Supplemental Table S7. Demographics of the Unmatched Study Cohort

	M-PCR/P-AST	SUC	
	Mean (SD)		p-value
Full sample (n = 423 M-PCR/P-AST; n = 133 SUC)	1.43 (0.53)	2.89 (1.15)	<0.0001
Sample of age 60+ (n = 320 M-PCR/P-AST; n = 81 SUC)	1.45 (0.54)	2.94 (1.13)	<0.0001

Supplemental Table S8. Comparison of Turnaround Times between M-PCR/P-AST and SUC

	M-PCR/P-AST	SUC	
	Mean (SD)		p-value
Full sample (n = 329 M-PCR/P-AST; n = 55 SUC)	1.51 (0.55)	3.53 (1.14)	<0.0001
Sample of age 60+ (n = 253 M-PCR/P-AST; n = 43 SUC)	1.51 (0.55)	3.50 (1.10)	<0.0001

Supplemental Table S9. Comparison of Turnaround Times between M-PCR/P-AST and SUC for All Matched Subjects with Positive Results

	Overall		
	SUC	M-PCR/P-AST	*p*-value (SUC vs. M-PCR/ P-AST)
	n/total (%)	*n*/total (%)	
Recurrence of UTI symptoms	32/148 (21.6%)	69/429 (16.1%)	0.13327
Medical Provider Visit (for UTI)	49/148 (33.1%)	93/429 (21.7%)	0.00769
Hospital, ER, or UC Visit (for UTI)	22/148 (14.9%)	34/429 (7.9%)	0.02299
All Negative Outcomes	67/148 (45.3%)	139/429 (32.4%)	0.0054

Supplemental Table S10. Comparison of Negative Outcomes for All Unmatched Subjects

	≥ 60 Years of Age		
	SUC	M-PCR/P-AST	*p*-value (SUC vs. M-PCR/ P-AST)
	n/total (%)	*n*/total (%)	
Recurrence of UTI symptoms	22/91 (24.2%)	48/325 (14.8%)	0.03968
Medical Provider Visit (for UTI)	29/91 (31.9%)	69/325 (21.2%)	0.04962
Hospital, ER, or UC Visit (for UTI)	17/91 (18.7%)	20/325 (6.2%)	0.00061
All Negative Outcomes	42/91 (46.2%)	98/325 (30.2%)	0.00564

Supplemental Table S11. Comparison of Negative Outcomes for Unmatched Subjects ≥ 60 Years of Age

Total (*N* = 577)	SUC Arm (*n* = 148)	M-PCR/P-AST Arm (*n* = 429)	*p*-value
Not Treated with Antimicrobial Agents (*n*, %)	49, 33.1%	132, 30.8%	
Treated with Antimicrobial Agents (*n*, %)	99, 66.9%	297, 69.2%	0.6
Empirical Treatment (*n*, %)	66, 66.7%	84, 28.3%	
Directed Treatment (*n*, %)	33, 33.3%	127, 71.4%	<0.0001

Supplemental Table S12. Comparisons of percentages of patients empirically or directedly treated with antimicrobial agents among the unmatched subjects in the SUC and M-PCR/P-AST arms

Total (*n* = 416)	SUC Arm (*n* = 91)	M-PCR/P-AST Arm (*n* = 325)	*p*-value
Not Treated with Antimicrobial Agents (*n*, %)	22, 24.2%	99, 30.5%	
Treated with Antimicrobial Agents (*n*, %)	69, 75.8%	226, 69.5%	0.24
Empirical Treatment (*n*, %)	48, 69.6%	67, 29.7%	
Directed Treatment (*n*, %)	21, 30.4%	159, 70.4%	< 0.0001

Supplemental Table S13. Comparisons of percentages of patients empirically or directedly treated with antimicrobial agents among the unmatched subjects $\geq$ 60 in the SUC and M-PCR/P-AST arms
